# Comprehending expository texts: the dynamic neurobiological correlates of building a coherent text representation

**DOI:** 10.3389/fnhum.2013.00853

**Published:** 2013-12-12

**Authors:** Katherine Swett, Amanda C. Miller, Scott Burns, Fumiko Hoeft, Nicole Davis, Stephen A. Petrill, Laurie E. Cutting

**Affiliations:** ^1^Education and Brain Sciences Research Lab, Peabody College of Education and Human Development, Vanderbilt UniversityNashville, TN, USA; ^2^Department of Psychology and Neuroscience, Regis UniversityDenver, CO, USA; ^3^Department of Psychiatry, University of California San FranciscoSan Francisco, CA, USA; ^4^Department of Psychology, Ohio State UniversityColumbus, OH, USA

**Keywords:** discourse processing, expository text comprehension, situation model building, temporal analysis of text comprehension, central vs. peripheral information, fMRI BOLD, semantic control network

## Abstract

Little is known about the neural correlates of expository text comprehension. In this study, we sought to identify neural networks underlying expository text comprehension, how those networks change over the course of comprehension, and whether information central to the overall meaning of the text is functionally distinct from peripheral information. Seventeen adult subjects read expository passages while being scanned using functional magnetic resonance imaging (fMRI). By convolving phrase onsets with the hemodynamic response function (HRF), we were able to identify regions that increase and decrease in activation over the course of passage comprehension. We found that expository text comprehension relies on the co-activation of the semantic control network and regions in the posterior midline previously associated with mental model updating and integration [posterior cingulate cortex (PCC) and precuneus (PCU)]. When compared to single word comprehension, left PCC and left Angular Gyrus (AG) were activated only for discourse-level comprehension. Over the course of comprehension, reliance on the same regions in the semantic control network increased, while a parietal region associated with attention [intraparietal sulcus (IPS)] decreased. These results parallel previous findings in narrative comprehension that the initial stages of mental model building require greater visuospatial attention processes, while maintenance of the model increasingly relies on semantic integration regions. Additionally, we used an event-related analysis to examine phrases central to the text's overall meaning vs. peripheral phrases. It was found that central ideas are functionally distinct from peripheral ideas, showing greater activation in the PCC and PCU, while over the course of passage comprehension, central and peripheral ideas increasingly recruit different parts of the semantic control network. The finding that central information elicits greater response in mental model updating regions than peripheral ideas supports previous behavioral models on the cognitive importance of distinguishing textual centrality.

## Introduction

Reading comprehension is a complex process that requires the coordination and integration of a number of component cognitive skills. The ability to read single words in isolation is widely accepted as one skill critical to comprehension, but successful reading comprehension does not always directly stem from adequate word identification skills. Some individuals who are skilled word readers are not skilled passage comprehenders (e.g., Cain and Oakhill, 2006; Catts et al., 2006; Cutting et al., 2009), supporting the idea that reading comprehension requires processes above and beyond single word reading.

Theoretical models of reading comprehension propose that successful comprehension requires a reader to draw on both text-based information and prior knowledge in order to build a coherent and meaningful mental representation of the text (Kintsch, 1974; van den Broek, 1988; Gernsbacher, 1990; Graesser et al., 1994; Zwaan and Singer, 2003). This mental representation is the reader's understanding of the text's deeper meaning; it consists of ideas from the text, relevant background knowledge, and inferences the reader makes about things not explicitly stated in the text (McNamara and Magliano, 2009). Building this mental representation is a dynamic process because cognitive demands change over time. For example, readers are known to spend more time processing words and sentences at the beginning of a text relative to later points (Glanzer et al., 1984). This could be due to the fact that, without context or relevant background knowledge activated to facilitate comprehension, comprehension necessitates more effortful attention to the initial construction of a mental representation (Yarkoni et al., 2008). Conversely, later stages of comprehension processes are facilitated by an increasing semantic contextualization (McNamara and Kintsch, 1996; McNamara and Magliano, 2009).

A number of imaging studies have examined the neurobiological correlates of reading comprehension (e.g., Fletcher et al., 1995; Maguire et al., 1999; Xu et al., 2005; Speer et al., 2009). Patterns of activation emerge when processing discourse that cannot be predicted from models of reading single words, or even single sentences, in isolation (Xu et al., 2005). Areas that consistently appear to be unique to processing narrative texts include the dorsal medial prefrontal cortex and bilateral temporal parietal junction, often attributed to social cognition required in story comprehension, bilateral temporal poles (TP, see Table 1 for all abbreviations), which play a role in generating specific semantic associations in connected text, and posterior medial structures, including posterior cingulate cortex (PCC) and precuneus (PCU), which have been associated with updates in and integration of the reader's mental model (e.g., St. George et al., 1999; Robertson, 2000; Gernsbacher and Kaschak, 2003; Yarkoni et al., 2008; Speer et al., 2009; Whitney et al., 2009; Price, 2012). This demonstrates that reading connected text involves additional processes beyond the phonological, orthographic, semantic, and syntactic processes seen at the word and sentence level. Still, many questions regarding how readers form a coherent text representation remain unanswered.

**Table 1:** **Abbreviations of neural regions**.

**Abbreviation**	**Region**
AG	Angular gyrus
DlPFC	Dorsolateral prefrontal cortex
IFG	Inferior frontal gyrus
IPL	Inferior parietal lobule
IPS	Intraparietal sulcus
ITG	Inferior temporal gyrus
MTG	Middle temporal gyrus
PCC	Posterior cingulate cortex
PCU	Precuneus
RSA	Retrosplenial cortex
SPL	Superior parietal lobule
STG	Superior temporal gyrus
STS	Superior temporal sulcus
TP	Temporal pole

Only a handful of studies have examined how the neural correlates of discourse processing change over the temporal progression of the discourse (Xu et al., 2005; Yarkoni et al., 2008; Speer et al., 2009). Of the few, Xu et al. (2005) used fMRI to compare the activation associated with reading the beginning of a story (setting and initial events) with the activation associated with reading the end of the story (outcome and final events). They found that processing the story's setting and initiating events resulted in strongly left lateralized activation, while processing the story's outcome resulted in increased activation in right hemisphere perisylvian and extrasylvian regions thought to contribute to inference and contextualization of narrative (Xu et al., 2005). These right hemisphere regions have since been related to social cognition processes that may be narrative-specific (Mar, 2011). This study provides evidence that reading comprehension not only involves processes distinct from those required in single word reading, but also that comprehension demands can vary from point to point within a given text.

Similarly, by modifying the cohesiveness of text (stories vs. scrambled sentences) Yarkoni et al. (2008) identified neural regions that showed linear increases in activation as a function of reading time. More specifically, they compared *construction* processes (i.e., those involved at the text outset as the reader lays a foundation for the mental representation) with *maintenance* processes (i.e., those involved in integrating new ideas onto previously read, related ones). They found that regions in the posterior parietal cortex associated with visuospatial updating and attention are involved in the construction of a reader's mental model, while perisylvian language areas were more involved in its maintenance. These studies support theoretical models that suggest that building a mental representation of text is a dynamic process in which the cognitive demands shift from one point in the text to the next.

Nevertheless, it is important to note that all of the aforementioned fMRI studies on discourse processing have exclusively examined narrative texts; none to date have examined expository texts (i.e., texts written to convey factual information on a topic). However, event-related potential (ERP) and behavioral studies suggest such genre distinctions are important. For example, Baretta et al. (2009) used ERP to distinguish between narrative and expository texts. They found that reading the final sentence of expository texts relative to narrative texts elicited a greater increase in N400 amplitude, and they concluded that expository texts required more demanding semantic processing. Eason et al. (2012) also reported differences between genres, showing that expository texts placed higher demands on executive function (EF) than narrative texts, particularly inferencing and planning/organizing information. EF is thought to be essential to the process of building a coherent text representation because it enables readers to store previously read text ideas as they simultaneously read new ideas and integrate them into their mental representation (Kintsch and Rawson, 2005). While behavioral data certainly support the theoretical significance of EF to reading comprehension in general (e.g., Carretti et al., 2005; Cain, 2006; Swanson et al., 2007; Cutting et al., 2009; Sesma et al., 2009; Locascio et al., 2010; Christopher et al., 2012), Eason et al.'s (2012) findings of higher demands on EF for expository text suggest that for this particular text genre, which is critical for acquiring new information, EF is particularly salient.

## Sensitivity to structural centrality

One hallmark of successful reading comprehension is that the reader can distinguish between ideas that are important, or central, to the overall meaning of the text, and those that are less important, or peripheral. Skilled readers form connections among a text's semantically related ideas as they read. The ideas and their connections form a network in the reader's mind (van den Broek and Espin, 2010). Some ideas are causally or logically connected to a great number of other ideas and as a result emerge as being important, or central, to the overall meaning of the text, while others have relatively few connections and fall out as being peripheral, or unimportant (Trabasso and van den Broek, 1985; van den Broek, 1988).

A robust finding in the comprehension literature is that skilled readers are more likely to recognize and recall an idea the more central it is to the overall meaning of the text (Kintsch et al., 1975; Kintsch and van Dijk, 1978; Britton et al., 1980; Cirilo and Foss, 1980; van den Broek, 1988). This finding holds for both narrative and expository texts (Miller and Keenan, 2011). van den Broek et al. (2013) propose that a reader's ability to distinguish a text's central and peripheral ideas, or their *sensitivity to structural centrality*, is an important indicator of their comprehension ability. For example, adults show greater sensitivity to structural centrality than do children (Brown and Smiley, 1977); typically-developing children show greater sensitivity to centrality than do children with reading disability (Miller and Keenan, 2009) as well as those with Attention Deficit Hyperactivity Disorder (ADHD) (Miller et al., 2013); and readers show greater sensitivity to centrality when reading in their native compared to foreign language (Miller and Keenan, 2011).

Importantly, studies suggest that centrality tends to emerge as a feature of the developing text representation. van den Broek (2012) used eye-tracking equipment to show that skilled readers fixate more frequently and spend more time reading central ideas than they do peripheral ones. This suggests that centrality is a dynamic construct that emerges as the reader processes a text, consistent with the idea that readers form connections among semantically related text ideas as they read. In theory, the ideas that are most important stand out because they are the ones that have the most connections and are consequently the ones most likely to be recalled. To date, although sensitivity to centrality has been investigated behaviorally, the neural mechanisms remain unknown. Understanding the neural mechanisms underlying sensitivity to centrality may allow for a more specific understanding of normal and disrupted comprehension processes.

## Current study

The current study sought first to identify the neural correlates specific to expository text comprehension, looking both at regions which overlap with single word processing and those which are specific to discourse-level processing. Once the systems for expository text comprehension were identified, we employed temporal analysis techniques to examine how these systems change over the course of building and maintaining a coherent mental representation of the text.

We hypothesized that when isolating discourse-level comprehension from word-level comprehension, we would see regions that have previously been implicated by sentence and narrative comprehension, particularly those associated with discourse-level language processing separate from social cognition [bilateral TP, angular gyrus (AG), and PCC] (Price, 2012; Chow et al., 2013). We predicted that the other traditional language regions, such as left-lateralized inferior frontal gyrus (IFG), middle temporal gyrus (MTG), and anterior superior temporal sulcus (STS), would most likely be shared by both word and passage tasks, but that these multi-functional regions would behave differently over the temporal course of passage comprehension compared to single-word comprehension (Chow et al., 2013). Additionally, due to prevalence of information organization in expository comprehension, we expected to see activations in the dorsal attention network [dorsolateral prefrontal cortex (dlPFC), intraparietal sulcus (IPS), inferior parietal lobule (IPL)], which has been associated with the kind of updating, integrating, and immediate planning of information that has been behaviorally described in previous studies on expository comprehension (Eason et al., 2012; Ptak, 2012).

We consequently hypothesized that over the course of passages alone, semantic control areas shared by words and passages at the mean-level would become increasingly responsive over time in passage comprehension alone due to the increased semantic demands associated with integrating and maintaining new information in a global text representation. Given Yarkoni et al.'s (2008) study showing that the parietal visuospatial attention regions are involved in the *construction* of text (and building the necessary visuospatial representation), while classic language areas (perisylvian language areas) are reflective of the *maintenance* of readers' mental models, we hypothesized that along with the emergence of a greater reliance on perisylvian regions over time, in passages we would see a decrease over time of posterior parietal regions.

The second goal of the current study was to examine the patterns of neural activation that are uniquely associated with processing central vs. peripheral ideas. While behavioral measures clearly indicate that readers distinguish central from peripheral ideas, both online and when recalling the text, the neurobiological processes that support this fundamental aspect of text comprehension have yet to be explored. Gaining insight into processes that promote a reader's sensitivity to centrality advances current comprehension models. More focally, it allows for isolating the underlying neural mechanisms supporting processes that may be disrupted in individuals with poor sensitivity to centrality. Such knowledge may eventually inform ways to individualize intervention for problematic reading comprehension. Given previous behavioral findings, we expected there to be unique semantic and integrative regions that differentiate central ideas from peripheral ideas. Finally, we predicted that with the temporal progression of the text, there are changes in the cognitive demands required in differentiating central and peripheral information and integrating those units into the mental model, resulting in temporally dynamic neural systems for different types of textual information.

To accomplish the goals of the study, an fMRI passage comprehension task was designed in which participants viewed three types of stimuli: (a) coherent expository passages (*Passages* condition) and (b) scrambled words (*Words* condition) and (c) non-alphanumeric symbols (*Baseline* condition). Within the Passages condition, we delineated the text's central and peripheral ideas. To examine differences between central vs. peripheral ideas, as well as overall patterns of activation associated with text, we employed a typical general linear model (GLM). To examine the emergence of a mental representation of the text, or dynamic changes taking place over time, an approach sensitive to temporal features was taken (Grill-Spector et al., 2006), whereby examination of neural activation that emerged or diminished over time for various conditions was revealed.

## Methods

### Participants

Seventeen adults (mean 24.7 years ± 3.3 years; 9 male) participated in the study. All participants met the following inclusion criteria: (1) native English speakers; (2) normal hearing and vision; (3) no history of major psychiatric illness; (4) no traumatic brain injury/epilepsy; (5) no history of a developmental disability; and (6) no contraindication to MRI. Each participant gave written consent at the beginning of the study, with procedures carried out in accordance with Vanderbilt University's Institutional Review Board. All participants had a standard score within the average range (85–115) on a composite of standardized reading tests (Sight Word Efficiency and Phonemic Decoding Efficiency subtests of Test of Word Reading Efficiency; Word Identification and Word Attack subtests of the Woodcock Reading Mastery Test-Revised) or had no history of difficulty with reading. Participants received $25 as compensation for a 2-h testing session.

### fMRI tasks

#### Passages condition (see Figure 1A)

Coh-Metrix 2.0 (Graesser et al., 2004) was used to create 8 passages that were equivalent across measures of word concreteness, syntactic simplicity, referential cohesion, causal cohesion, and narrativity. Passages were matched on descriptive factors, including: number of words, average sentence length, and all passages measured a Flesch-Kincaid grade-level between 4.0 and 4.9. To insure that passages were equivalent in difficulty, each of the 8 passages was isolated and compared to the mean of the remaining 7 passages. Passages were considered equivalent when measures were within a 90% confidence interval of the mean of the group of remaining passages. At the end of this process, the passages were equal across 23 measures of descriptive statistics, vocabulary frequency, word concreteness, syntactic simplicity, referential cohesion, causal cohesion, and narrativity (i.e., the degree to which the text uses everyday oral conversation and tells a story with familiar characters, events, places, and things). Four of these passages were used for the Passages condition and four were used for the Words condition (see below), which included words from the passages in randomized order.

All passages were 150 words in length. Each sentence was no longer than 13 words. The passages were all expository and included the following topics: Hang Gliding, Wrasses, Velvet Worms, and Hydroponics. Each passage consisted of two paragraphs, the first of which served to introduce the topic while the second elaborated on a particular detail of the subject matter.

#### Defining centrality

Consistent with established procedures for determining central vs. peripheral ideas (Albrecht and O'Brien, 1991; Miller and Keenan, 2009, 2011), centrality of the passages' idea units was defined using importance ratings obtained from a sample of 14 adult volunteer raters using the following procedure. First, the raters read one of the four passages related to Hydroponics, Wrasses, Hang Gliding, and Velvet Worms. Next, we presented that passage to the rater, this time formatted as a checklist of all the ideas in the passage. Each idea on the checklist corresponded with a phrase presented in-scanner. Raters used this checklist to identify how important each idea was to the overall meaning of the passage using a 0–7 Likert scale that ranged from the idea being “unimportant to the passage” to “very important to the passage.” We calculated a mean rating for each idea unit. The ratings formed a normal distribution and had high reliability estimates (ICCs: Hydroponics = 0.90; Wrasses = 0.88; Hang Gliding = 0.89; Velvet Worms = 0.91). Consistent with previous work (Miller and Keenan, 2009, 2011), we used a median-split to divide this distribution of idea units into two classes; we identified ideas below the median rating as “peripheral” and those above the median as “central.”

Previous studies suggest that the neural correlates of reading words can vary according to grammatical class (e.g., nouns vs. verbs; see Vigliocco et al., 2011, for a review) or conceptual concreteness (Kiehl et al., 1999; Wang et al., 2010). To rule out such potential confounds, we examined whether central and peripheral ideas were comparable in the number and types of nouns and verbs they contained. Within each of the four passages, we used *t*-tests to assess whether central and peripheral ideas significantly differed on the number of action verbs, non-action verbs, abstract nouns, concrete nouns, or pronouns. Central and peripheral ideas did not significantly differ on any of these classifications [*Hydroponics t*_(55)_ = −1.04, *p* = 0.30; *Wrasses t*_(50)_ = −0.96, *p* = 0.34; *Hang gliding t*_(43)_ = −0.47, *p* = 0.64; *Velvet worms t*_(48)_ = −1.34, *p* = 0.19], abstract nouns [*Hydroponics t*_(55)_ = −0.87, *p* = 0.39; *Wrasses t*_(50)_ = −1.61, *p* = 0.11; *Hang gliding t*_(43)_ = −1.36, *p* = 0.18; *Velvet worms t*_(48)_ = 0.00, *p* = 1.00], action verbs [*Hydroponics t*_(55)_ = −1.00, *p* = 0.32; *Wrasses t*_(50)_ = 1.31, *p* = 0.20; *Hang gliding t*_(43)_ = −0.38, *p* = 0.71; *Velvet worms t*_(48)_ = −1.26, *p* = 0.22], or non-action verbs [*Hydroponics t*_(55)_ = 1.79, *p* = 0.08; *Wrasses t*_(50)_ = −1.41, *p* = 0.17; *Hang gliding t*_(43)_ = 1.59, *p* = 0.12; *Velvet worms t*_(48)_ = −1.22, *p* = 0.23].

#### Words baseline

**Figure 1:**
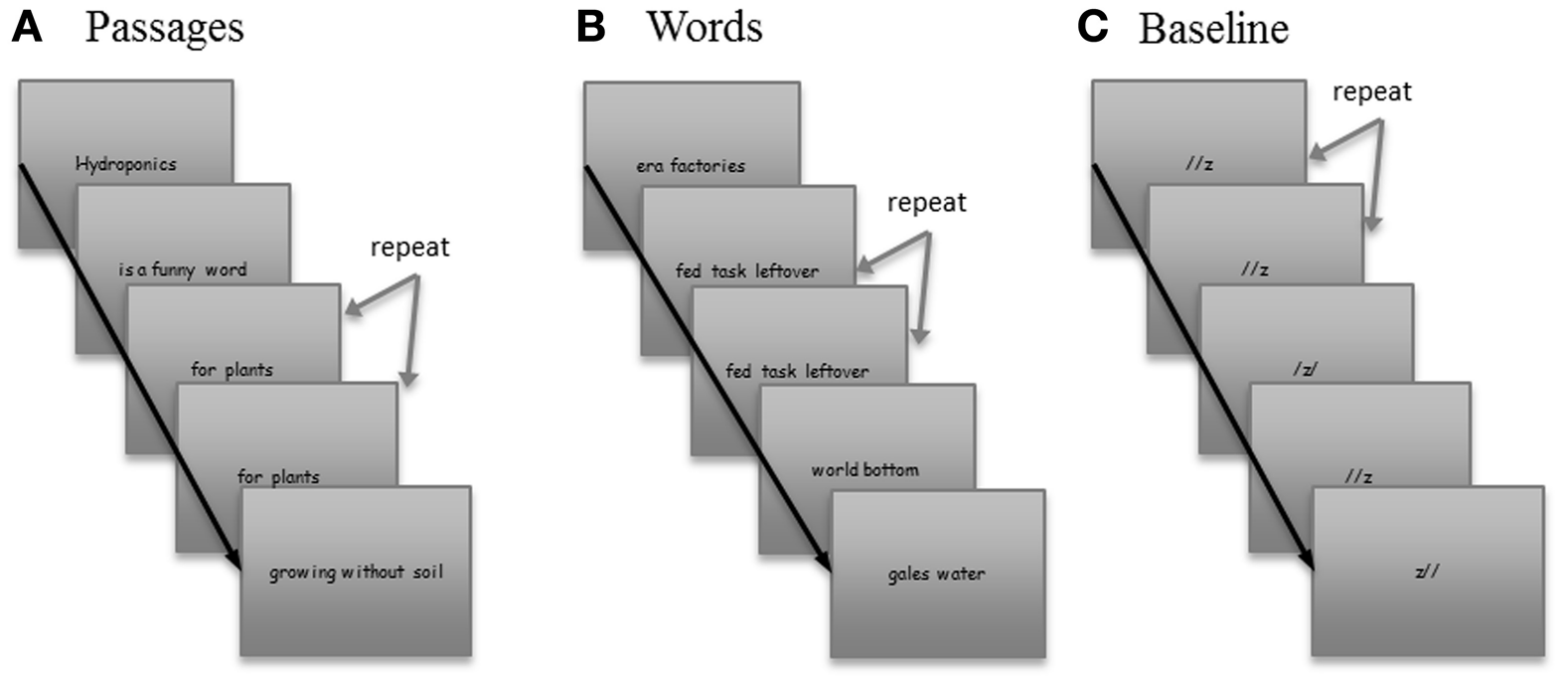
**Sample stimuli from each of the three conditions**. Stimuli consisted of three conditions, **(A)** Passages, **(B)** Words, and **(C)** Baseline. Order of three conditions was defined by two order lists which were randomly administered to subjects.

The words baseline condition consisted of scrambled words presented in “phrases,” which were exactly matched in length, word type, and presentation time to the phrases in the passages (see Figure 1B).

#### Baseline

The baseline condition included three non-alphanumeric symbols displayed horizontally on a slide (see Figure 1C).

### Procedure

Using imaging technology to explore the neural correlates of reading comprehension is a challenging task due to the temporal nature of discourse processing. Previous studies have presented the entire paragraph on one screen (Fletcher et al., 1995; Vogeley et al., 2001; Moss et al., 2011), but this procedure prohibits comparing how readers process specific aspects of the passage, such as central vs. peripheral ideas, because the block contains both types of information.

The most temporally precise presentation method is to present the story one word at a time, and several studies have employed this procedure (Xu et al., 2005; Yarkoni et al., 2008; Speer et al., 2009). When piloting passages using this approach, participants reported that it created an uncomfortable, artificial reading experience, likely in part because readers typically process words up to 14–15 letters to the right of their fixation (Rayner, 1986), and using a single word-by-word presentation prevents this. The moving window procedure is an alternative method that allows examination of the processing associated with single words. The advantage of this procedure is that the word(s) immediately preceding and following the word under fixation are also visible. Although this allows for a more naturalistic reading experience, the approach was undesirable for this study because it requires a self-paced design, and temporal consistency in the presentation of stimuli is required for group comparisons.

To avoid both the above confounds, we presented our passages one meaningful phrase at a time. This procedure enabled us to compare activation related to processing central and peripheral ideas, yet decreased the artificial demands imposed by a word-by-word presentation. Each phrase was presented on a separate trial. The phrases included noun phrases, verb phrases, and prepositional phrases, and they ranged from 1 to 6 words in length. The number and type of words presented together determined the phrases' presentation duration. We allowed 550 ms for each content word and 275 ms for each function word. For timing purposes, we presented no more than three content words per slide and randomized the time between phrases to allow comparison across phrases. The Words condition followed the same presentation format as the Passages condition. The baseline condition was presented between paragraph 1 and paragraph 2 of both the Passages and Words conditions. The purpose of this design was to allow participants' activation to return to baseline after reading each block (paragraph). The presentation sequence was: (1) Passage condition, Paragraph 1; (2) Baseline condition; (3) Passage condition, Paragraph 2; (4) Baseline condition; (5) Words condition; (6) Baseline condition. The mean time for the passages block was 78.54 (*SD* = 22.94); Baseline mean = 47.69 (*SD* = 1.48); and Words mean = 82.45 (*SD* = 3.29).

In all three conditions, 8% of the stimuli were repeated on two consecutive screens. To monitor whether participants attended to the stimuli, participants pressed a button with their right thumb when they detected a phrase repetition or a symbol configuration repetition. Mean percentage correct response was very high (95.06 ± 5.36).

### fMRI data acquisition, preprocessing, and first-level analyses

Imaging was performed on a research-dedicated Philips Achieva 3T MR scanner with a 32-channel head coil. Functional images were acquired using a gradient echo planar imaging sequence with 40 (3 mm thick) slices with no gap and consisted of 4 runs, each 7 min (190 dynamics per run). Other relevant imaging parameters for the functional images are *TE* = 30 ms (for optimal BOLD contrast at 3T), FOV 240 × 240 mm, slice thickness = 3 mm with 0 mm gaps, 75° flip angle, *TR* = 2200 ms, and a matrix size 80 × 80 (interpolated), implying 3 mm^3^ isotropic voxels.

All functional data were analyzed using MATLAB (Mathworks, Natick, MA) and SPM8 (Frackowiak et al., 1997). The functional data for each participant were slice-timing corrected, aligned to the mean functional image, normalized to MNI space, and spatially smoothed with a 8 mm FWHM Gaussian filter. Participants whose data in any run exceeded motion thresholds (>3 mm translational displacement, 3° rotation) were discarded from the analysis. First-level analysis was performed by creating a standard regression model with estimated HRF for each condition while the six motion parameters (x, y, z translational; x, y, z rotational) and outlying volumes as determined by ART (Whitfield–Gabrieli; http://www.nitrc.org/projects/artifact_detect/) added to the design matrix as regressors of no interest.

For the standard GLM analyses (i.e., those examining mean group-level activation, heretofore referred to as “mean group-level analyses”), three sets of contrasts for each participant were created. First, we compared total activation for the Passages against the Baseline condition as well as the Central and Peripheral conditions against the Baseline condition. Then, Central – Baseline and Peripheral – Baseline contrasts were directly compared. Finally, to further understand the potential overlap and specificity of passages as related to scrambled words, we examined the Boolean conjunction of Passages – Baseline and Words – Baseline (see Supplementary data).

To investigate the dynamic processes involved in building a coherent text representation, brain regions were examined that demonstrated increased or decreased activation as a function of time as the participant progressed through the Passages, relative to the Baseline.

These temporal analyses determined whether the dynamic process of building a text representation was associated with increased or decreased activation in specific areas. To accomplish these analyses, for each run we modeled each phrase onset as a stick function with a height equal to the difference between phrase onset and the initial phrase of Paragraph 1. For instance, to examine increased activation over time for the Central phrases, if Central phrases were presented at the 3rd, 7th, and 9th TR, the resulting vector would be [0 0 1 0 0 0 5 0 7]. The “1” in the vector represents the onset in which the first event of interest (i.e., central or peripheral phrase) occurs. Any proceeding event is weighted in proportion to the time passed between the new event and original event of interest. The resulting vectors were then convolved using the HRF to create conditions of interest and were inserted into the first-level GLM. This formulation allowed us to model a linear relationship, thus representing temporal/dynamic changes associated with each condition. To temporally model the Passages against the baseline, we collapsed the Central and Peripheral onset vectors and built the condition of interest using the same formulation above.

### Group-level imaging analysis

SPM8 and MATLAB (Mathworks, Nattick, MA) were used to create whole brain activation maps. Individual contrast maps were brought up to a group level one-sample *T*-test to analyze the Passages, Peripheral phrases, and Central phrases relative Baseline, and the Central and Peripheral phrases relative to each other. MNI coordinates were converted to Talairach (Talairach and Tournoux, 1988) using formulas by Matthew Brett (http://www.mrc-cbu.cam.ac.uk/) and locations were determined by querying the Talairach Daemon (Lancaster et al., 2000). The group-level analyses were subjected to a uncorrected statistical threshold of *p* < 0.001 and a cluster size of 90 voxels, which was determined by 3dClustSim to be equivalent to *p* < 0.05.

## Results

### Passages vs. baseline: mean group-level analysis

**Figure 2:**
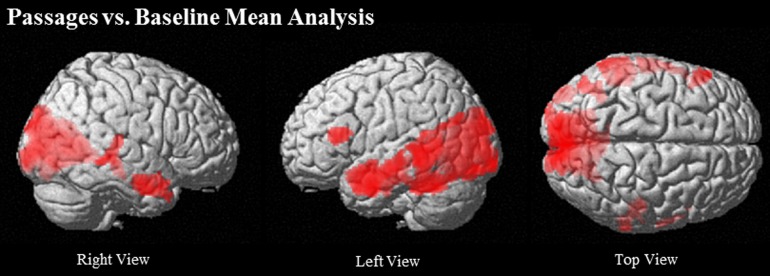
**Regions that show greater activation in Passages than Baseline at (uncorrected) *p* < 0.001, *k* = 90**.

Passages relative to baseline showed robust activation of traditional language regions, specifically left IFG (BA 45/46), left MTG (21/22) extending to AG (BA 39), left TP, and bilateral anterior STS. Additionally, PCC and ventral PCU were active, along with visual and word-processing regions, including bilateral occipital, fusiform, lingual gyri, and cuneus clusters (see Table 2 and Figure 2).

**Table 2:** **Passages vs. Baseline mean analysis**.

**Mean contrast**	**Anatomical region**	**Talairach coordinates**			**Cluster size**	**Stat max *T***	***BA***
		***x***	***y***	***z***			
Passages > Baseline	LH Lingual Gyrus	−9	−82	0	8234	10.34	18
	LH Cuneus	−14	−77	6	[]	9.88	17
	RH Lingual Gyrus	12	−88	−2	[]	8.61	18
	RH PCC	2	−55	6	[]	8.51	30
	LH Cerebellum	−14	−77	−16	[]	7.92	*
	RH Cerebellum	6	−50	0	[]	7.13	*
	LH PCC	−5	−68	10	[]	7.04	30
	RH Cuneus	6	−82	16	[]	6.78	18/17
	LH Culmen/Fusiform	−40	−48	−23	3337	8.01	37
	Gyrus						
	LH ant MTG	−47	−3	−20	[]	7.15	21
	LH Parahippocampal	−23	−27	−6	241	6.43	28/27
	LH IFG/MFG	−53	23	20	163	6.01	45/46/9
	RH MTG	47	−30	−1	280	5.75	22/21
	RH anterior MTG	55	2	−16	421	5.55	21/38
	RH Amygdala	21	0	−17	[]	4.96	*
	LH Angular Gyrus	−44	−73	24	141	5.17	39
	RH Parahippocampal	19	−25	−7	105	5.06	28/35
	Gyrus						

Cluster size in mm^3^. BA, Brodmann Area. All T-values are significant at p = 0.05.

* Indicates region outside of Brodmann areas.

For large clusters, brackets indicate sub-cluster peaks in BA regions distinct from primary peak, extracted using a decreased peak search space of 4 mm within the main cluster.

### Passages vs. baseline: temporal group-level analyses

#### Temporal – increasing

**Figure 3:**
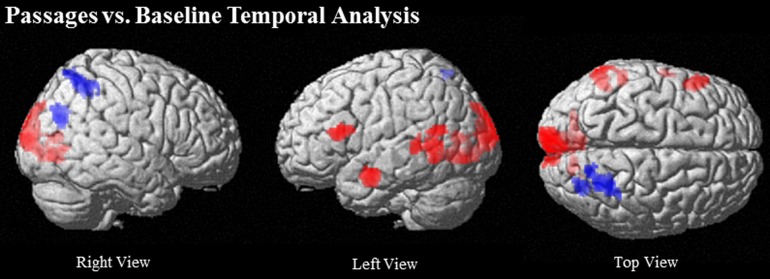
**Regions that increase over time (red) and decrease over time (blue) in Passages vs. Baseline at (uncorrected) *p* < 0.001, *k* = 90**.

Comparison of the passages condition to baseline elicited increasing activation over time predominantly in left hemisphere language related regions, including left IFG (BA 44/45), left MTG (BA 21/22), and left anterior STS. Bilateral lingual gyrus (BA 18) extending to cuneus and left occipital also showed increasing activation (see Table 3 and Figure 3).

**Table 3:** **Passages vs. Baseline temporal analysis**.

**Temporal contrast**	**Anatomical region**	**Talairach coordinates**			**Cluster size**	**Max *T***	***BA***
		***x***	***y***	***z***			
Passages > Baseline							
Increasing	LH Lingual Gyrus	−14	−72	−6	1197	7.99	18
	RH Cuneus	4	−92	5	[]	7.69	17
	LH Cuneus	−9	−91	8	[]	6.46	17
	LH IFG	−49	16	19	140	6.84	44/45
	LH ant STS	−53	−6	−12	151	6.39	21
	LH STG	−51	−39	7	268	5.46	22
	LH AG	−53	−55	14	[]	5.16	39
	LH MTG	−60	−54	2	[]	5.14	21
	RH Lingual Gyrus	10	−74	−3	150	5.46	18
Decreasing	RH dorsal PCU/IPS	24	−55	50	300	6.75	7
	RH PCU	26	−70	23	156	6.75	31

Cluster size in mm^3^. BA, Brodmann Area. All T-values are significant at p = 0.05.

For large clusters, brackets indicate sub-cluster peaks in BA regions distinct from primary peak, extracted using a decreased peak search space of 4 mm within the main cluster.

#### Temporal – decreasing

Comparison of the passages condition to baseline indicated prominent decrease in activation over time in right dorsal PCU extending to IPS (BA 7) (see Table 3 and Figure 3).

### Central and peripheral comparisons: mean group-level analyses

#### Central and peripheral ideas compared to baseline

As to be expected, both the Central and Peripheral ideas from the Passages condition showed robust bilateral occipital activity and greater language (left IFG, left MTG, left AG, bilateral anterior STS, left TP) and PCC activity than right when compared to the baseline (see Table 4).

**Table 4:** **Central and peripheral mean analysis**.

**Mean contrast**	**Anatomical region**	**Talairach coordinates**			**Cluster size**	**Max *T***	***BA***
		***x***	***y***	***z***			
Central > Peripheral	LH Cuneus	−3	−79	20	12206	11.29	17
	LH Cerebellum	−12	−72	−10	[]	10.01	*
	LH Lingual	−11	−82	2	[]	9.87	18
	RH Lingual	1	−86	0	[]	9.40	18
	RH PCU	2	−69	18	[]	8.78	31
	LH Parahippocampal Gyrus	−18	−50	−3	[]	8.74	28
	RH PCC	2	−65	14	[]	8.60	31
	LH PCC	−20	−64	7	[]	8.17	31
	RH Cuneus	10	−77	8	[]	7.15	17
	RH Cerebellum	16	−46	−7	[]	6.71	*
	LH ant STS	−49	8	−14	427	9.00	38/21
	LH SPL/Postcentral	−15	−53	62	191	6.79	5/7
	RH SPL/PCU	11	−50	57	90	5.22	7
Central > Baseline	LH Cuneus/Lingual	−2	−83	23	25104	16.50	18/19
	RH STG	43	−25	4	899	6.01	13/22
	RH MTG	60	−42	−8	[]	5.94	20
	LH Postcentral Gyrus	−52	−9	46	164	5.87	4/3
Peripheral > Baseline	LH Lingual Gyrus	−20	−65	0	13057	10.33	19/18
	LH IFG/MFG	−51	27	22	541	7.03	45/9
	RH STG/MTG (STS)	45	−29	−3	509	6.64	22
	RH MTG/ITG	38	−2	−30	391	6.34	21/20
	LH Postcentral	−52	−9	46	172	5.73	3
	LH Parahippocampal Gyrus	−23	−25	−6	135	5.33	28
	LH Hippocampus	−27	−11	−19	129	5.23	28
	RH Cerebellum	33	−37	−22	126	5.20	*

Cluster size in mm^3^. BA, Brodmann Area. All T-values are significant at p = 0.05.

* Indicates region outside of Brodmann areas.

For large clusters, brackets indicate sub-cluster peaks in BA regions distinct from primary peak, extracted using a decreased peak search space of 4 mm within the main cluster.

#### Comparison of central vs. peripheral ideas

**Figure 4:**
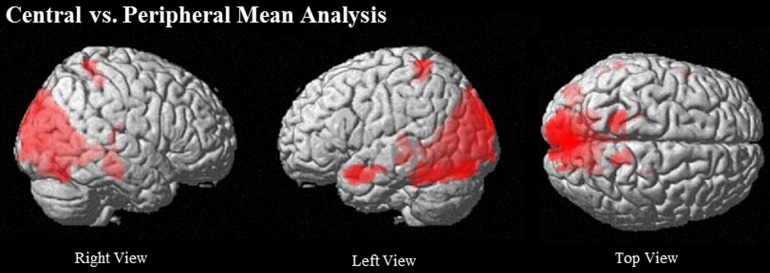
**Regions that show greater activation when reading central compared to peripheral ideas at (uncorrected) *p* < 0.001, *k* = 90**.

Directly comparing Central to Peripheral, the Central condition showed more activation in posterior mid-line structures, including retrosplenial cortex (RSA) (BA 29), PCC (BA 31), and ventral and dorsal PCU clusters (BA 31 and BA 7), along with a large cuneus cluster that extended into lingual gyrus (BA 18/19). Laterally, Central showed greater activation in left TP and left anterior STS (BA 38 and 21) (see Table 4 and Figure 3). Peripheral compared to Central phrases did not show significantly greater activation in any region (see Table 4 and Figure 4).

### Peripheral and central phrase comparisons: temporal group-level analyses

#### Central and peripheral phrases compared to baseline—increasing

**Figure 5:**
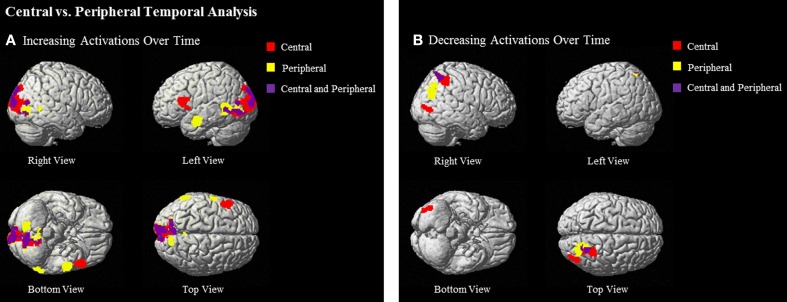
**Regions that show (A) increasing and (B) decreasing activations over time of central ideas (red), peripheral ideas (yellow), and both central and peripheral ideas (purple) at (uncorrected) *p* < 0.001, *k* = 90**.

Central ideas as compared to baseline showed significantly greater increase in activation over time in many of the same areas that were recruited to a greater extent over time during the passages vs. baseline conditions: left IFG (BA 44/45), left cuneus (17/18), and left lingual gyrus (18/19). Similarly, temporal group-level analyses examining increases in activation for peripheral ideas also elicited activation in left MTG (BA 21), left cuneus (BA 19/18), bilateral lingual gyrus (BA 18), and left superior temporal gyrus (STG) (BA 21/22) (see Table 5 and Figure 5A).

**Table 5:** **Central and peripheral temporal analysis**.

**Temporal contrast**	**Anatomical region**	**Talairach coordinates**			**Cluster size**	**Max *T***	***BA***
		***x***	***y***	***z***			
Central v Baseline							
Increasing	LH IFG	−49	16	19	208	8.11	44/45
	LH Cuneus	−7	−92	6	853	6.77	17/18
	LH Lingual Gyrus	−12	−70	−6	299	6.49	18/19
Decreasing	RH PCU/SPL	26	−53	45	258	6.41	7
	RH Middle Occipital Gyrus	36	−81	3	91	5.98	18/19
Peripheral v Baseline							
Increasing	LH Cuneus	−9	−90	26	233	6.39	19/18
	RH Lingual Gyrus/Cerebellum	12	−70	−6	183	6.16	18
	LH MTG	−56	−6	−12	235	5.77	21
	LH MTG/STG	−60	−51	6	135	5.54	21/22
	LH Lingual Gyrus	−14	−72	−5	260	4.95	18
Decreasing	RH PCC/Cuneus	28	−69	20	215	7.18	31/18
	RH PCU/SPL	20	−57	50	98	4.95	7

Cluster size in mm^3^. BA, Brodmann Area.

All T-values are significant at p = 0.05.

#### Central and peripheral phrases compared with baseline—decreasing

Central phrases as compared to baseline in the decreasing temporal analyses elicited activation in right PCU and superior parietal lobule (SPL) (BA 7) and right middle occipital gyrus (18/19). Temporal analyses for peripheral phrases also showed significant decreasing activation in right PCU and SPL (BA 7) (see Table 5 and Figure 5B).

#### Comparison of central vs. peripheral phrases—increasing and decreasing

No statistically significant differences were found for either the increasing or decreasing temporal group-level analyses.

## Discussion

The neural correlates of expository text comprehension have not previously been examined in fMRI. This study not only sought to identify a network of regions specifically activated for discourse-level processing of expository text but, due to the fluctuating cognitive demands within the comprehension of a single text, also examined the neural systems that underlie comprehending the text over time. Finally, to further identify core processes of expository text comprehension, this study aimed to define the functional underpinnings of comprehending textual centrality, which is one key indicator that a reader has formed a coherent mental representation (van den Broek et al., 2013).

### Neural correlates of discourse processing in expository texts

In expository text comprehension, we see co-activation of left-lateralized language regions (IFG, posterior and anterior MTG) and two heteromodal association areas—left AG and PCC/PCU—commonly associated with higher-order cognitive processes (Price, 2012; Chow et al., 2013). Specifically, the observed language regions have been identified as part of an executive semantic control network (Whitney et al., 2011), with left IFG and left posterior MTG thought to direct semantic connections to fit the current context, while regions in the anterior temporal lobe are thought to store and integrate specific semantic associations (Binder et al., 2011; Whitney et al., 2011; Price, 2012). These regions have been observed to activate for different levels of comprehension (Price, 2012; Chow et al., 2013), and an examination of the scrambled words condition compared to expository comprehension in our own study shows that regions in this executive semantic control network overlapped for both conditions (see Supplementary Figure 1). From the perspective of hierarchical comprehension, in which reading is comprised of discourse comprehension built on top of single word comprehension, these shared regions could be interpreted as contributing to word-level processes only. However, given previous findings indicating these regions are active when processing semantic associations for words and sentences, hierarchical assumptions of functionality may overlook these regions' complex contributions to reading (Xu et al., 2005; Binder et al., 2011; Price, 2012; Chow et al., 2013). This complexity is supported by the temporal analyses discussed below, which show activations of the semantic control network over time that are unique to expository text.

The heteromodal regions that we see co-activated with the semantic control network (left AG and PCC/PCU) were activated for expository text, not words (see Supplementary Figure 1). The distinction of these regions as discourse-specific is unsurprising. Both regions have been previously identified as multi-function, cognitive “hubs,” which perform higher-order cognitive processes (Chow et al., 2013; Seghier, 2013). In the context of language, left AG is primarily associated with semantic memory, incorporation of semantic information into a coherent whole, and making top-down semantic predictions (Price, 2012; Seghier, 2013), while PCC has been noted for its activation at updates in readers' mental representation of narrative texts (i.e., where readers are required to integrate information that conflicts with the present situation model) (Maguire et al., 1999; Speer et al., 2009; Whitney et al., 2009). This co-activation of left AG and PCC, along with language regions suggests that expository text comprehension involves a core semantic-processing network which integrates semantic information both at the word- and sentence-level, along with activation of heteromodal regions that more globally update the situation model into a coherent whole. Further discussion of the PCC and PCU roles in the context of centrality can be found in the following section.

Our findings of posterior midline and left AG in expository text when compared to single word reading is similar to what is reported for narrative, and further supports the possibility that these regions are involved in global comprehension processes which aren't necessarily dependent on discourse type (Mar, 2011). Unlike previous findings on narrative comprehension, however, it's important to note that apart from these left-lateralized activations, our findings suggest that expository text comprehension does not rely on additional regions within the theory of mind network— a network associated with social inference processes and contextualization of narrative text within world knowledge (Xu et al., 2005; Ferstl et al., 2008; Mar, 2011). The absence of other primary hubs of the theory of mind network, particularly the medial prefrontal cortex, emphasizes that narrative and expository texts may have critically different cognitive requirements, stressing the need to examine both text types in order to isolate specific comprehension processes susceptible to dysfunction. A direct study of narrative and expository texts is needed to further explore these comparisons.

Contrary to our hypothesis, expository text did not show activation of the dorsal attention network. This could be a result of the fact that our participants were skilled adult readers, and our passages were written at a fourth grade reading level. We created the passages to be highly cohesive, easily decodable, and thus easy to comprehend. However, it is likely that these relatively undemanding passages decreased the overall EF load.

### Temporal dynamics of expository text comprehension

Interestingly, regions in the executive semantic control network progressively activate over time in passages alone, despite being activated in both passages and words in the mean analysis (See Supplementary Figure 2). This shows that these semantic regions have a unique activation pattern in expository text comprehension, further supporting findings that they play multi-functional roles interacting with different comprehension levels (Xu et al., 2005; Ferstl et al., 2008; Mar, 2011). Observations of the BOLD signal in these regions and its correlation to the HRF for central or peripheral events suggest that these increases are specifically due to language processes (see Supplementary Figures 5–7). During discourse comprehension, in order to maintain the reader's situation model, these semantic networks would necessarily be increasingly relied on over the course of the passage. As the amount of required semantic connections increases, both a greater store of semantic associations (eliciting activation in anterior MTG) and increased executive direction of those associations (left IFG and left posterior MTG) are required to ensure that new information aligns with and is integrated into the current situation model (Yarkoni et al., 2008; Whitney et al., 2011). It has also been suggested that left IFG and left posterior MTG play a role in integrating modality-specific knowledge (i.e., perceptual, motor, and affective) into the reader's situation model, which could also contribute to its increasing activation through comprehension (Chow et al., 2013).

When looking at regions that decrease over the course of comprehension, we see decreased activation of right IPS in both word and passage conditions (See Supplementary Figure 3). However, compared to words and baseline, the BOLD signal in right IPS shows a marked decrease in activation at central and peripheral events, suggesting that IPS could have a unique relationship with discourse-specific processes (see Supplementary Figure 7). Interestingly, this region has been previously implicated in discourse-level narrative comprehension. Ferstl et al. (2005) suggested that right IPS is involved in attentional shifts from local to global aspects of the mental representation of the text. In narrative comprehension compared to scrambled sentences, Yarkoni et al. (2008) saw an initial spike of activation in the same region, followed by a linear decrease over the time course of comprehension, attributing the activation pattern to visuospatial updates involved in initial situation model construction. Similarly, when contrasting the first paragraph of expository text to the second paragraph (see Supplementary Figure 4), we see activation in the same region, suggesting that right IPS is more prevalent in the beginning of comprehension than the end. Consequently, decreased activation of right posterior parietal cortex could be indicative of the region's role in construction of the situation model. The overlapping temporal decrease in scrambled words could reflect readers' initial attempts to build a situation model despite incoherence, particularly since task types were not identified to readers ahead of the stimuli. However, higher-order interpretations of IPS activations in texts should be treated carefully, as activations could reflect subtle, visual attention differences between tasks.

These findings closely reflect Yarkoni et al.'s (2008) narrative findings, and support a cross-genre reading model in which visuospatial updating and attention regions are involved in the initial construction of a reader's mental representation of a text, and executive semantic control areas are increasingly necessary for its maintenance. The similarities between studies suggest not only that there are distinct cognitive stages during text comprehension, but that some of the neural structures underlying these stages may be shared across text genre.

### Neural correlates of central and peripheral text

Our second aim was to examine the neural correlates associated with the ability to distinguish between a text's central and peripheral ideas, or readers' sensitivity to centrality (van den Broek et al., 2013). Skilled readers demonstrate sensitivity to centrality by recognizing and recalling a greater proportion of central than peripheral ideas (Kintsch et al., 1975; Kintsch and van Dijk, 1978; Britton et al., 1980; Cirilo and Foss, 1980; van den Broek, 1988); however, identifying central information is a skill known to be particularly vulnerable to disruption among individuals who experience comprehension difficulties (Miller and Keenan, 2009, 2011; Miller et al., 2013). Because sensitivity to centrality is both a critical component of comprehension and one that is vulnerable to disruption, we aimed to explore the neural underpinnings of this process.

A direct comparison of mean group-level activation indicates that central text ideas are cognitively distinct from peripheral ideas, eliciting greater activation in textual integration regions when compared to peripheral. Specifically, reading central relative to peripheral ideas was associated with posterior midline structures, namely PCC and PCU (BA 29/31), as well as anterior temporal regions. These findings relate to previous studies of discourse processing that have found PCC and PCU to be associated with forming connections among text ideas (Fletcher et al., 1995; Maguire et al., 1999; Robertson, 2000), updating story representations (Whitney et al., 2009), and connecting text-based information to prior knowledge (Fletcher et al., 1995; Maguire et al., 1999). Additionally, Speer et al. (2009) found greater PCC activation when readers processed the points in the text that required the greatest degree of mental model updating. Activation of STG/MTG (BA 38) has also been associated with linking semantic ideas to form a connected narrative (Fletcher et al., 1995; Maguire et al., 1999). These findings confirm that in addition to readers' ability to behaviorally distinguish between central and peripheral information, the degree of textual relevancy is associated with a distinct neural network of textual/extra-textual integration and mental representation regions in the comprehender.

### Temporal dynamics of central and peripheral text

Comparing central and peripheral activations over time shows that as the text progresses, central ideas recruit different parts of the language network than peripheral ideas. Specifically, regions within the executive semantic control network differentiate central and peripheral processing over time, with central ideas increasingly relying on the left IFG, and peripheral ideas activating left anterior MTG independently from and posterior MTG to a greater extent than central ideas. This centrality-driven division between frontal and temporal semantic processing regions can be seen in the BOLD signal, with left IFG and left anterior MTG initially responding generally to the switch from non-word to word stimulus, before demonstrating clear correlation with central and peripheral HRF prediction peaks, respectively (see Supplementary Figures 5, 6). While both temporal and frontal regions are implicated in semantic cognition, it has been suggested that left posterior MTG acts as a general interface between lexical and conceptual knowledge, anterior MTG is involved in specific semantic associations, while left IFG is more context-specific, activating for conceptual knowledge that is cued by the preceding text (Price, 2012; Chow et al., 2013). Consequently, for central textual ideas, which are more semantically-dependent on previous ideas, the IFG is increasingly involved in making appropriate semantic connections to the established context. On the other hand, processing peripheral ideas, or ideas which have looser semantic connections to the preceding text, would rely more heavily on regions that support general semantic knowledge to contextualize the present text. This suggests that within the fronto-temporal semantic control network, there is a functional divide between frontal and temporal contributions related to perception of textual centrality.

Decreased activation over time for both central and peripheral ideas was similar to the patterns of temporal activations associated with passages—as language regions increased over time, activation of the visuospatial attention system decreased. This pattern is also apparent in the BOLD signal, and appears to be anti-correlated with both central and peripheral phrases (see Supplementary Figure 7). However, the extent and strength of the right IPS cluster in central ideas was significantly greater than peripheral. This difference can be explained by right IPS involvement in situation model construction—because central ideas contribute more to the situation model, they would consequently be more sensitive to the decreasing need of construction regions (Kintsch, 1988).

## Limitations and future directions

Our temporal analyses assumed a linear relationship between time and neural activation of text processing; however, non-linear temporal relationships may exist, and future studies should explore such non-linear changes. A second limitation is that our models assume that neural activation builds not only as the reader progresses through the paragraphs, but also during the baseline condition between the two paragraphs. Future work should compare whether removing this baseline assessment changes the patterns of temporal activation change.

One methodological consideration is that our participants were skilled adult readers, and our passages were written at a fourth grade reading level. Future studies should manipulate the reading level of the passages and examine how this manipulation influences the neural correlates of expository comprehension, particularly regions associated with EF.

Future studies should also consider the important interaction between text and reader by considering the background knowledge that the readers hold about each passage topic. Background knowledge plays an important role in building a coherent representation of the text (Spilich et al., 1979; Miller and Keenan, 2009, 2011) and allows the reader to form a more meaningful representation that goes beyond the text-based ideas (Kintsch, 1988; Albrecht and O'Brien, 1991). A reader's existing knowledge base is especially important to consider with respect to expository texts because they often use topic-relevant vocabulary that builds upon the reader's assumed knowledge base.

Finally, future work should examine the neural correlates of building a coherent text representation among groups of readers known to be less sensitive to structural centrality, such as individuals with reading disability, individuals with ADHD, and foreign language learners. Comparing the patterns of activation associated with skilled and less skilled comprehension could help identify the comprehension processes that are disrupted and the underlying source of their comprehension difficulties. This insight could perhaps be employed to inform and improve reading comprehension instruction and interventions.

## Conclusion

Successful expository text comprehension is critical in school learning environments and requires different cognitive processes than narrative comprehension, including a greater ability to organize and plan information to develop a cohesive mental representation (Eason et al., 2012). Expository text consequently offers a unique environment to study reading ability and disability. However, while there is an increasing number of imaging studies examining discourse processing through narrative comprehension, expository text comprehension has largely been overlooked. This study not only identifies the neural correlates of expository text comprehension as a whole, but also isolates those regions involved in the dynamic construction of mental representations of the text over time, as well as those associated with textual centrality. By better understanding these dynamic, within-text processes of reading comprehension, we can begin to identify the key cognitive stages of comprehension that are particularly prone to dysfunction in populations with discourse-level reading disabilities.

### Conflict of interest statement

The authors declare that the research was conducted in the absence of any commercial or financial relationships that could be construed as a potential conflict of interest.
