# Supplementary material for: Comprehending expository texts: the dynamic neurobiological correlates of building a coherent text representation
Source: Front Hum Neurosci. 2013 Dec 12;7:853. doi: 10.3389/fnhum.2013.00853 (PMC3860184; doi:10.3389/fnhum.2013.00853)
Supplement: Supplementary file 1 [file DataSheet1.ZIP › 64782_Cutting_Suppl Table_1.pdf]

Supplementary Table 1

Words vs. Baseline Mean Analysis

Mean Contrast	Anatomical Region	Talairach coordinates			Cluster Size	Max T	BA
		x	y	z			
Words > Baseline	LH CB	-42	-50	-25	18897	16.63	*
	LH Fusiform/Middle Occipital Gyrus	-22	-87	-12	[]	15.16	18
	RH Middle Occipital Gyrus/Fusiform	21	-87	-9	[]	12.86	18
	LH Lingual	-7	-82	-2	[]	11.62	17
	RH Lingual	8	-84	-2	[]	8.80	17
	LH MTG/STG	-58	-25	-5	[]	7.96	21
	RH STG	51	7	-12	1242	5.77	38
	RH MTG	49	-20	-6	[]	5.61	21
	RH Thalamus	19	-24	2	[]	5.57	*
	RH Hippocampus	27	-14	-11	[]	4.97	*
	RH IFG	36	17	-17	[]	3.77	47
	LH Postcentral Gyrus	-52	-11	47	368	7.62	3
	LH Precentral Gyrus	-42	-14	35	[]	6.05	4
	RH Postcentral Gyrus	52	-18	49	97	5.25	1/3
	RH Precentral Gyrus	54	-9	42	[]	4.17	4
	LH IFG	-49	15	23	185	5.24	45/9

Note. Cluster size in mm^3. BA = Brodmann Area. All *T* values are significant at $p = .05$. For large clusters, brackets indicate sub-cluster peaks in BA regions distinct from primary peak, extracted using a decreased peak search space of 4 mm within the main cluster.
