# Supplementary material for: Comprehending expository texts: the dynamic neurobiological correlates of building a coherent text representation
Source: Front Hum Neurosci. 2013 Dec 12;7:853. doi: 10.3389/fnhum.2013.00853 (PMC3860184; doi:10.3389/fnhum.2013.00853)
Supplement: Supplementary file 1 [file DataSheet1.ZIP › 64782_Cutting_Suppl Table_2.pdf]

Supplementary Table 2

*Words vs. Baseline Temporal Analysis*

| Mean Contrast | Anatomical Region | Talairach coordinates | | | Cluster Size | Max T | BA |
|---|---|---|---|---|---|---|---|
| | | x | y | z | | | |
| Word v Baseline | | | | | | | |
| Increasing | RH CB | 8 | -62 | -14 | 298 | 7.68 | * |
| | RH Cuneus | 8 | -59 | 8 | [] | 4.41 | 30 |
| | RH Lingual | 17 | -60 | -1 | [] | 4.34 | 19 |
| | LH Inferior Occipital Gyrus | -34 | -88 | -15 | 995 | 7.30 | 18 |
| | LH CB | -45 | -52 | -23 | [] | 7.25 | * |
| | LH fusiform | -43 | -39 | -22 | [] | 6.73 | 37 |
| | LH Lingual | -12 | -94 | -10 | [] | 4.36 | 17 |
| | RH Inferior/Middle Occipital Gyrus | 30 | -93 | -8 | 275 | 5.79 | 18/19 |
| | LH CB | -5 | -67 | -17 | 240 | 5.65 | * |
| | LH Lingual | -16 | -65 | -4 | [] | 4.77 | 19 |
| | LH Calcarine Fissure/Cuneus | 0 | -78 | 10 | 182 | 4.93 | 18 |
| | LH PCC | -9 | -65 | 12 | [] | 4.43 | 30 |
| Decreasing | RH PCU | 19 | -62 | 47 | 473 | 7.42 | 7/19 |
| | RH IFG | 45 | 3 | 29 | 151 | 6.31 | 45/9 |

*Note.* Cluster size in $mm^3$. BA = Brodmann Area. All *T* values are significant at $p = .05$. For large clusters, brackets indicate sub-cluster peaks in BA regions distinct from primary peak, extracted using a decreased peak search space of 4 mm within the main cluster.
