# Supplementary figures and images for: Comprehending expository texts: the dynamic neurobiological correlates of building a coherent text representation
Source: Front Hum Neurosci. 2013 Dec 12;7:853. doi: 10.3389/fnhum.2013.00853 (PMC3860184; doi:10.3389/fnhum.2013.00853)

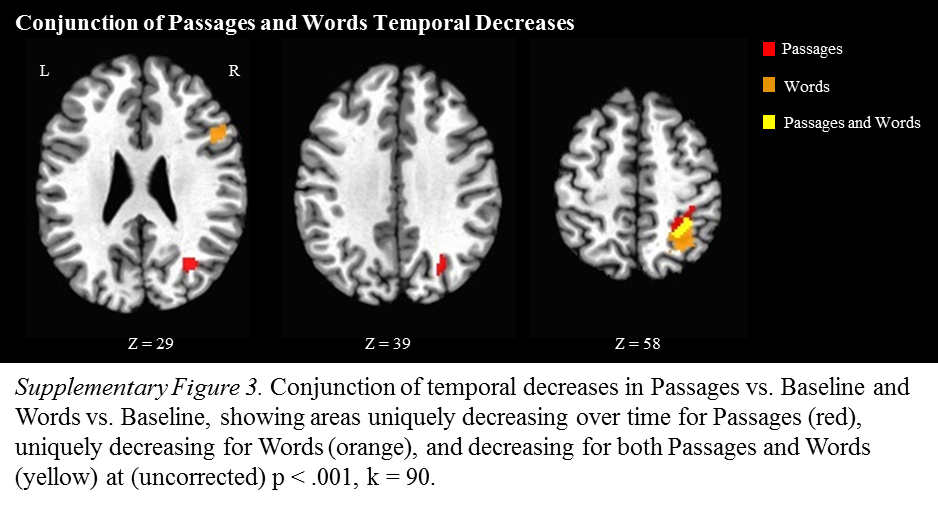

Supplement: Supplementary file 1 [file DataSheet1.ZIP › 64782_Cutting_Suppl_Figure_3.TIF]

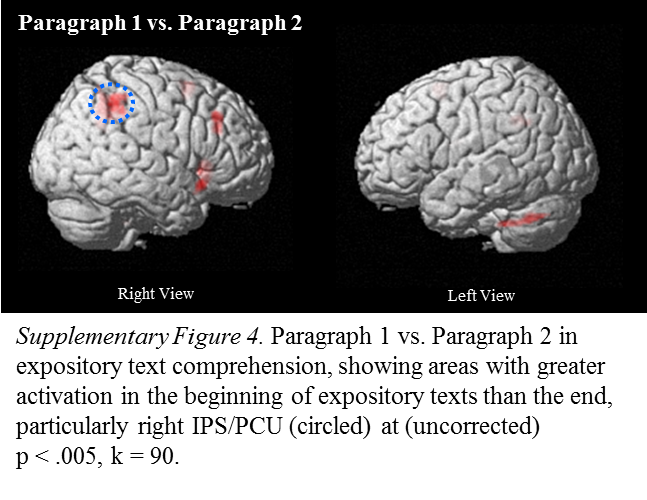

Supplement: Supplementary file 1 [file DataSheet1.ZIP › 64782_Cutting_Suppl_Figure_4.TIF]

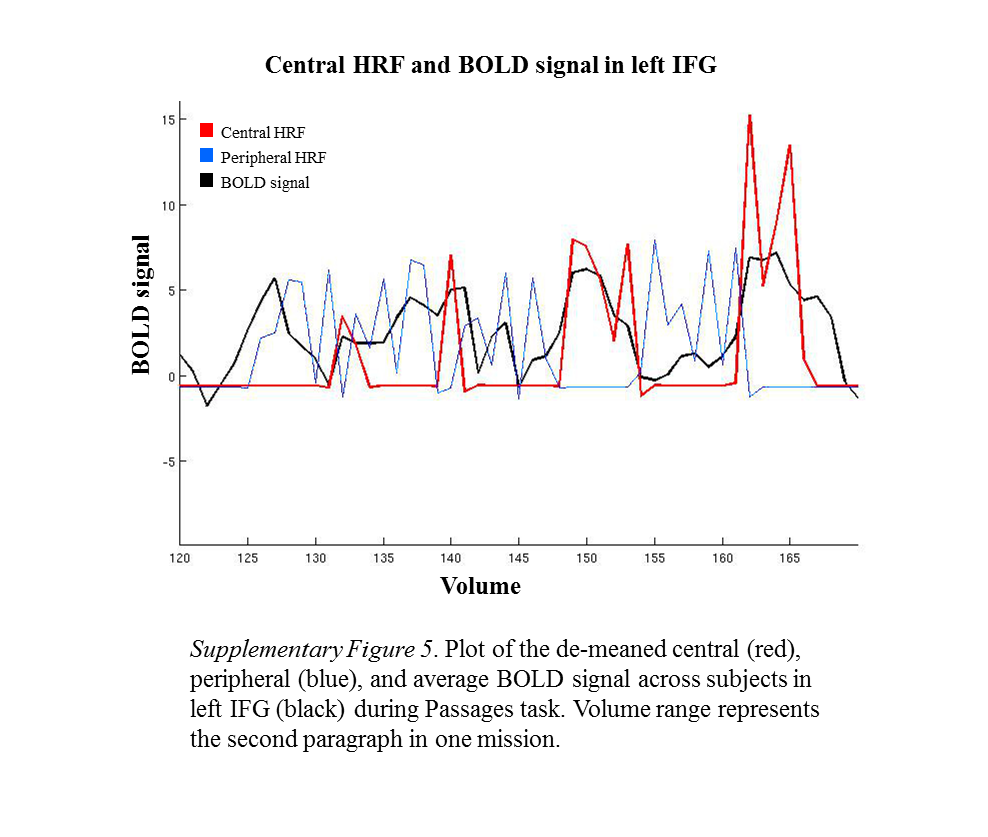

Supplement: Supplementary file 1 [file DataSheet1.ZIP › 64782_Cutting_Suppl_Figure_5.TIF]

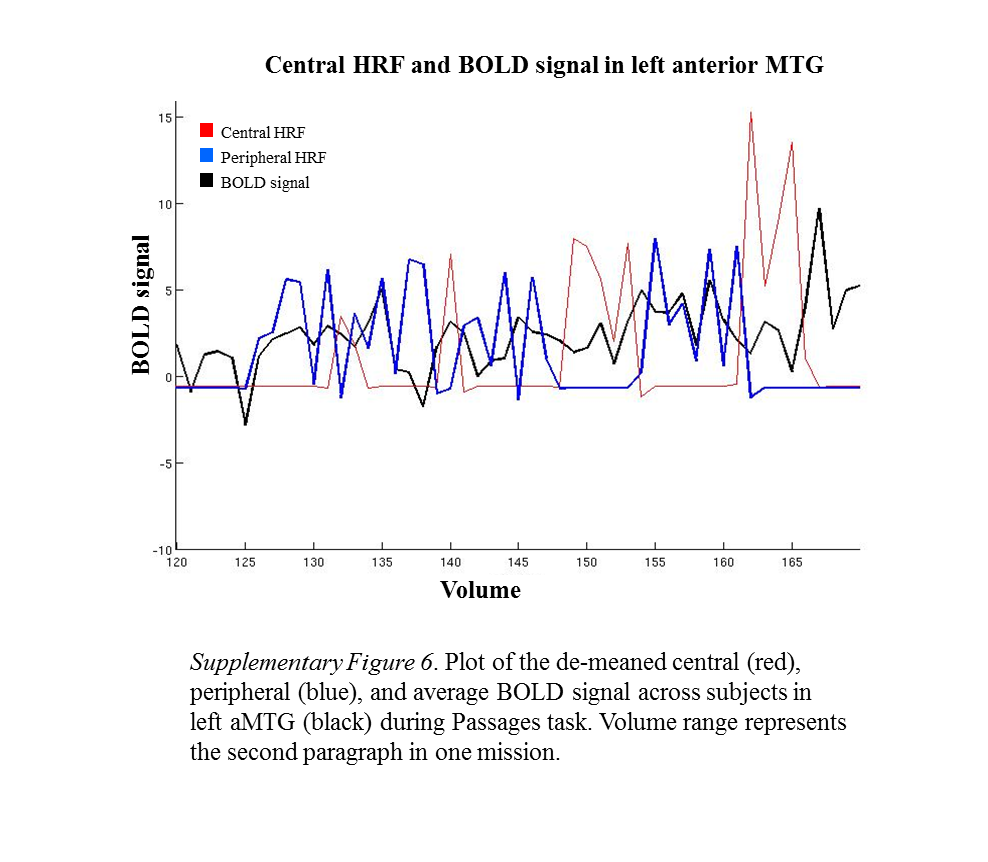

Supplement: Supplementary file 1 [file DataSheet1.ZIP › 64782_Cutting_Suppl_Figure_6.TIF]

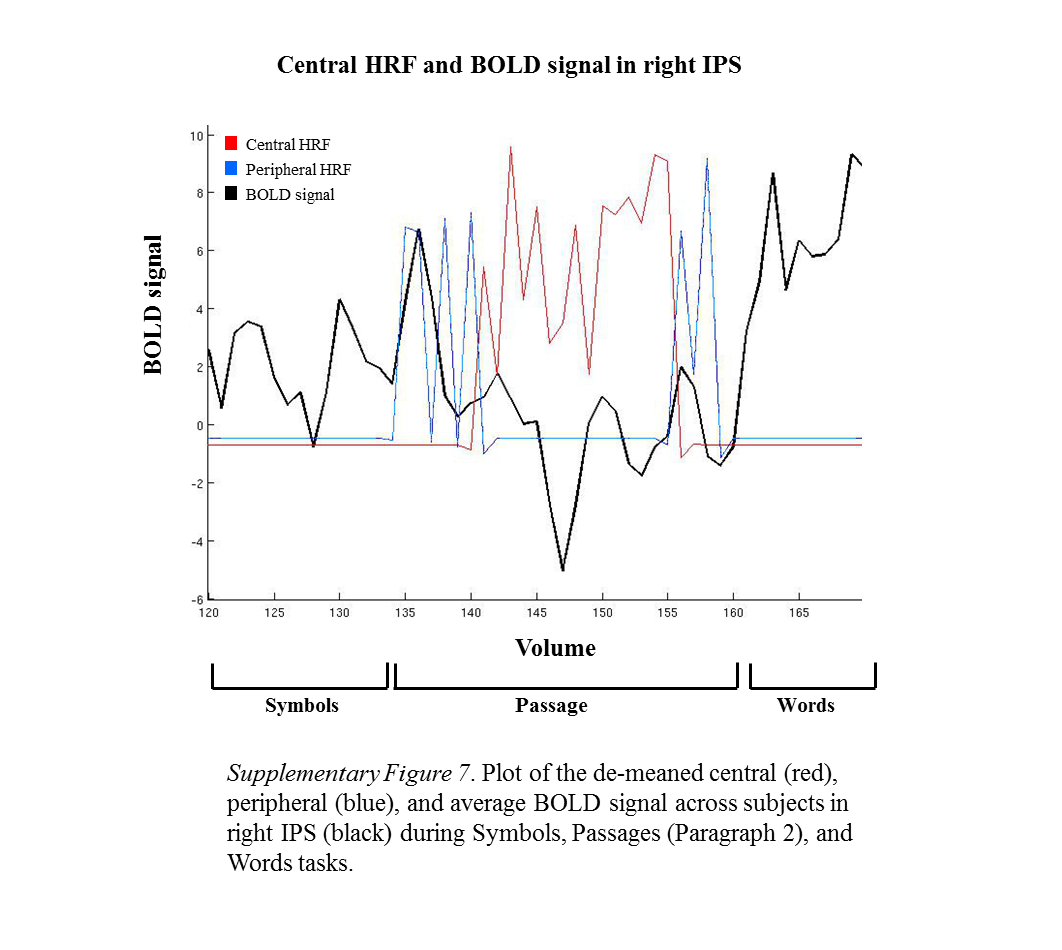

Supplement: Supplementary file 1 [file DataSheet1.ZIP › 64782_Cutting_Suppl_Figure_7.TIF]

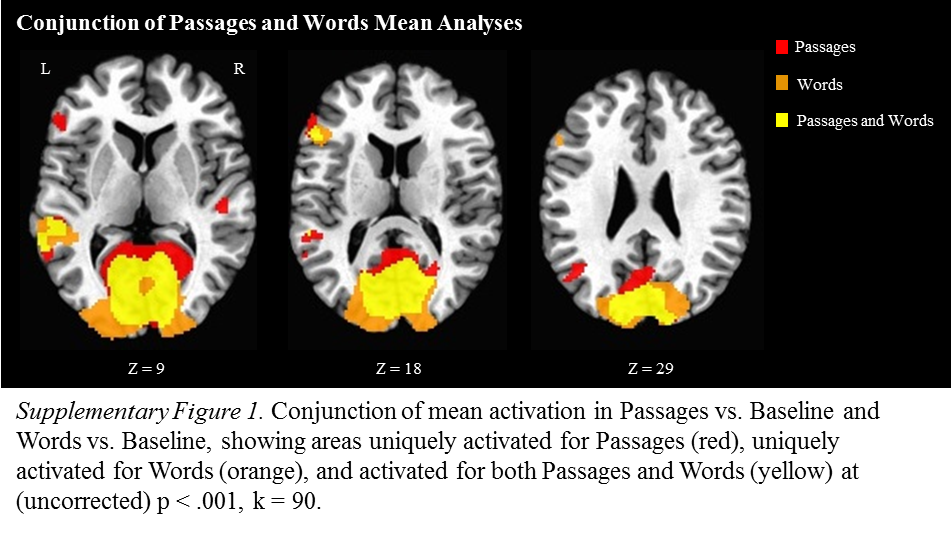

Supplement: Supplementary file 1 [file DataSheet1.ZIP › 64782_Cutting_Suppl_Figure_1.TIF]

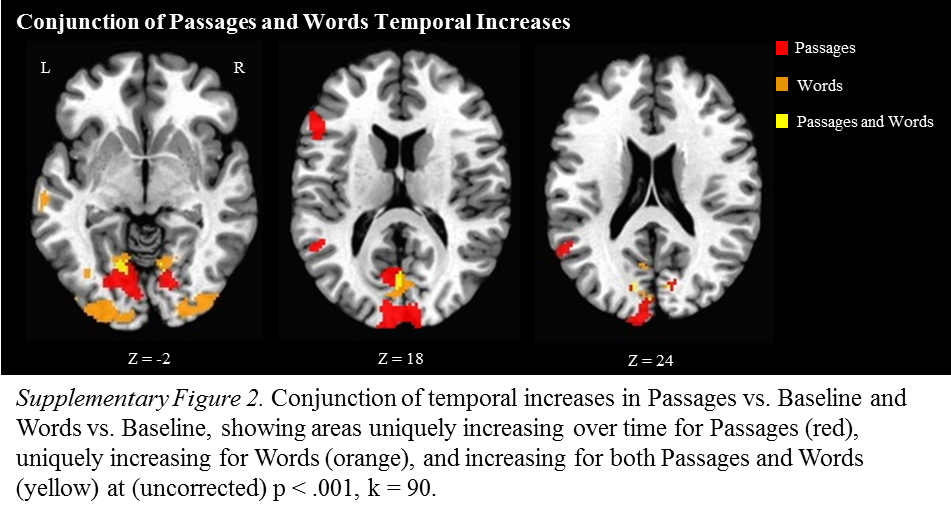

Supplement: Supplementary file 1 [file DataSheet1.ZIP › 64782_Cutting_Suppl_Figure_2.TIF]
